# Metastatic Sarcomatoid Carcinoma of the Larynx: A Case Report

**DOI:** 10.7759/cureus.70331

**Published:** 2024-09-27

**Authors:** Jihane Derfoufi, Fatima Rezzoug, Ouissam Al Jarroudi, Sami Aziz Brahmi, Said Afqir

**Affiliations:** 1 Medical Oncology, Mohammed VI University Hospital, Faculty of Medicine and Pharmacy of Oujda, Mohammed First University of Oujda, Oujda, MAR; 2 Medical Oncology, Mohammed VI University Hospital, Oujda, MAR

**Keywords:** cancer metastasis, female patient, oncological management, rare malignancy, sarcomatoid carcinoma of head-neck region

## Abstract

Laryngeal spindle cell carcinoma (SpCC) is a unique and very aggressive form of laryngeal cancer. This neoplasm, consisting of malignant epithelial and mesenchymal elements, presents major diagnostic challenges, and there is a lack of conventional therapeutic approaches due to its rarity. This report describes a case of a 53-year-old female who experienced persistent difficulty speaking and a dry cough after contracting coronavirus disease 2019 (COVID-19). Histological and immunohistochemical studies confirmed the diagnosis of sarcomatoid cancer. Initially, the patient received cisplatin and doxorubicin-based chemotherapy. However, the tumor's growth prompted a change in treatment to weekly paclitaxel. Despite initial signs of improvement, the patient experienced acute alveolar hemorrhage, and sudden and severe bleeding in the small air sacs of the lungs, which unfortunately led to her death while receiving treatment in the critical care unit.

The diagnosis and treatment of laryngeal SpCC pose significant challenges due to its uncommon and aggressive nature, and there is still no consensus on the most effective therapeutic approach. Existing therapies often incorporate modifications from other sarcoma regimes. Recent studies have demonstrated a possible positive reaction to immunotherapy, although further research is necessary to validate this. This report highlights the significant challenges of treating laryngeal sarcomatoid carcinomas and the importance of continued research to improve diagnostic and treatment options, especially given the absence of established guidelines.

## Introduction

Spindle cell (sarcomatoid) carcinoma (SpCC) of the larynx is an uncommon neoplasm, accounting for only around 2-3% of all laryngeal malignancies. Squamous cell carcinoma (SCC) is the most aggressive form of cancer in the larynx, and spindle cell tumor is a particularly dangerous subtype of SCC [1]. The 2017 World Health Organization Classification of Head and Neck Tumors highlights that SpCC is a kind of tumor that originates from a non-specialized stem cell and consists of both epithelial and mesenchymal components. Experts describe it as a monoclonal neoplasm. A study suggests that the unique spindle cell look of cancerous cells of SpCC can be attributed to the epithelial-mesenchymal transition [2]. We classify the tumor as biphasic, comprising an SCC and a mesenchymal-appearing malignant spindle cell component.

The malignant spindle cell component originated from a mutant epithelial clone. Histology commonly reveals a mixture of squamous and spindle cells, arranged in various patterns such as storiform, solid, and fascicular [3]. Diagnosing the tumor requires an immunohistochemical analysis of epithelial and mesenchymal markers, including AE1/AE3, epithelial membrane antigens (EMA), vimentin, desmin, and S-100. However, the results can vary. Ultrastructural investigations can supplement immunohistochemistry, a valuable diagnostic tool, to prevent false diagnosis [3]. The glottic area is the most prevalent location, followed by the supraglottic region. There is a substantial correlation between laryngeal SpCCs and alcohol and tobacco usage, and the majority of cases are reported in middle-aged to elderly males.

Dysphonia, hoarseness, and, less often, dyspnea are the most common symptoms at presentation when they emerge at the subglottic location. Since this condition is rare, there are no widely accepted treatment guidelines [4]. We present a case of metastatic laryngeal sarcomatoid carcinoma in a female patient. Our primary objective is to enhance the understanding of the clinical characteristics, diagnostic challenges, and therapeutic options associated with this uncommon pathology. We believe our findings will contribute to the existing literature by providing additional information about the clinical aspects and prospective outcomes of patients with laryngeal sarcomatoid carcinoma.

## Case presentation

The patient was a 53-year-old female with no significant previous medical history. She had experienced a coronavirus disease 2019 (COVID-19) episode four months before her admission, which had been characterized by dysphonia and a dry cough. Despite receiving therapy for her symptoms, there was no improvement. The clinical exam revealed that the patient had an Eastern Cooperative Oncology Group (ECOG) performance level of 1, stage III dyspnea according to the New York Heart Association (NYHA) staging method, and clean lymph nodes in the neck and distant chains. The general clinical examination revealed no abnormalities within the scope of the exploration.

**Figure 1:**
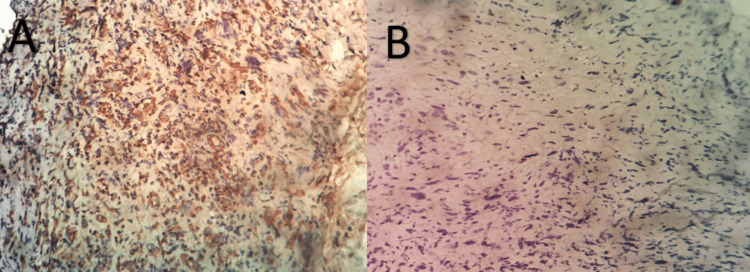
Immunohistochemical image of a laryngeal sarcomatoid carcinoma A) Diffuse positive anti-vimentin staining (clone V9; DAKO) in tumor cells (staining with hematoxylin at x40 magnification). B) Negative staining for anti-cytokeratin AE1/AE3, CK7, CK 5/6, P63, and desmin (staining with hematoxylin at x40 magnification)

**Figure 2:**
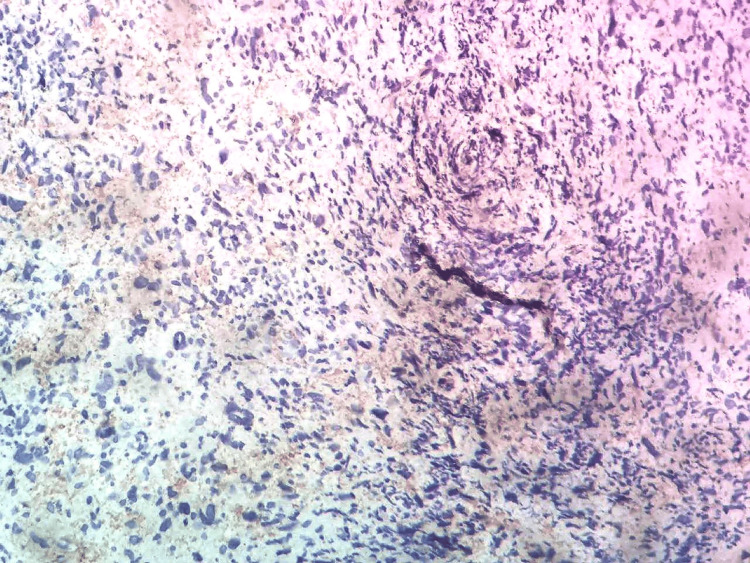
Immunohistochemical image showing positive staining of tumor cells with anti-AML Anti-AML (clone 1A4; DAKO). Staining with hematoxylin at x40 magnification

A laryngeal fibroscopy revealed a tiny polypoid growth on the front part of the right voice cord. The pathological examination revealed a tumor growth characterized by a high number of cells, which were very abnormal and had swollen, irregular, and occasionally variable nuclei. Intermittent fusiform appearances were seen. An immunohistochemistry test showed that the S100 protein was not present, but cytokeratin was. There was some expression of EMA, but none of the other markers - CD34, desmin, and CD30 - were present. The pathological criteria, which indicate an undifferentiated carcinomatous process of sarcomatoid type, led to the diagnosis (Figures 1-2).

**Figure 3:**
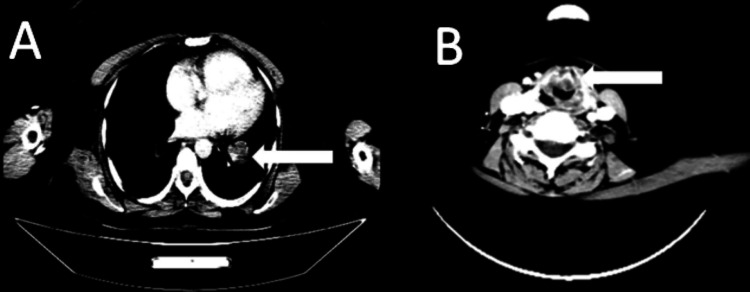
Horizontal CT scan image passing through the laryngeal tumor and the metastatic pulmonary lesion A) Infiltrative glottic laryngeal tumor process, 17 x 16 x 14 mm, affecting the anterior commissure and vocal cord. B) Proximal left lung secondary localization in the lower lobe, measuring 36 mm CT: computed tomography

More tests to determine the stage of the tumor showed that it was in the glottic area of the larynx, affecting the front parts of both vocal cords and the anterior commissure. The tumor, measuring 17 x 16 x 14 mm in its largest dimensions, had infiltrated the fat spaces in front of the throat. Despite its attachment to the base of the epiglottis, it did not exhibit any signs of spreading into the surrounding fat space or the area below the vocal cords. It was categorized as T2. Small lymph nodes with minor axes of less than 1 cm were identified in the IIA and IIB chains on both sides. These lymph nodes were nonspecific and did not surpass 9 mm in size. We classified the 36-mm-diameter pulmonary lesion in the left proximal lower lobe as M1 (Figure 3).

We identified a massive pulmonary embolism, which occupied almost the entire space within the main pulmonary artery and its branches, both near and far from the heart, and showed indications of pulmonary hypertension. We detected no more remote, suspicious lesions. We conducted a transthoracic biopsy on the pulmonary tumor, and the histological and immunohistochemical results confirmed the presence of sarcomatoid cancer. We diagnosed the patient with laryngeal sarcomatoid cancer that had spread to the lungs based on the clinical, radiological, and pathological information.

The multidisciplinary team (MDT) decided to focus the therapeutic approach on treating oligo-metastatic laryngeal sarcomatoid carcinoma, a specific type of cancer that had only spread to one area of the lungs. The therapeutic approach involved a treatment protocol that included cisplatin at a dosage of 70 mg/m^2^ and doxorubicin at a dosage of 50 mg/m^2^ on day one, with subsequent repetitions every 21 days. The patient's condition remained consistent and without any significant changes across six treatment cycles. The patient then underwent radiological monitoring. She experienced three episodes of febrile pancytopenia during her therapy, but we were unable to identify any specific infectious source. As a preventive measure, we administered 30 million units of G-CSF for five days following the treatment initiation.

Three weeks after the last treatment cycle, we observed a single instance of delayed neutropenia. A consultation with an internal medicine specialist was required. However, since the condition was resolving on its own, we did not conduct a bone marrow biopsy (BMB). A positron emission tomography (PET) scan to more accurately categorize the oligo-metastatic illness and evaluate potential curative therapy options revealed a lateralized glottic laryngeal lesion that had significant hypermetabolism, which was in line with the previously identified primary tumor. There were a small number of somewhat overactive cervical lymph nodes in the IIA areas on both sides. The histology revealed a hypermetabolic mass in the left para-hilar region of the lung, which matched the secondary site. There were hypermetabolic nodules in both lungs, with the largest one seen in the right para-hilar area, indicating that it may be a result of secondary involvement. We deemed an area of increased metabolic activity inside the bone marrow of the left humerus to be potentially concerning. Based on the PET scan findings, the medical team determined that the patient was not suitable for surgery and decided to monitor their condition.

After three months, the medical team conducted a further CT scan of the chest, abdomen, and pelvis (CTAP), revealing the tumor's advancement and the presence of a stable, significant pulmonary embolism with indications of pulmonary hypertension. The overall results suggested a negative progression of the tumor, as evidenced by chest discomfort, severe grade III dyspnea, and prolonged unresolved dysphonia. The MDT looked at the patient's history of treatment intolerance and pancytopenia episodes. They decided that, despite G-CSF support, limited data, and the lack of established therapeutic standards, the patient should start receiving 80 mg/m^2^ of paclitaxel once a week for three cycles before being reevaluated. The patient received three cycles of weekly paclitaxel, presenting a favorable tolerance profile and experiencing a reduction in dyspnea during the initial two rounds. Nevertheless, she had a sudden drop in oxygen levels, worsening her cough. A CT scan revealed alveolar bleeding and tumor progression. We brought the patient to the ICU, but sadly, she succumbed to alveolar hemorrhage within 24 hours.

## Discussion

SCC is the predominant kind of malignant tumor affecting the larynx. SpCC, or sarcomatoid carcinoma, is an extremely aggressive form of squamous cell cancer. This tumor is uncommon and has a documented occurrence rate of 2-3% among all cases of laryngeal malignancies [1]. The uterus and urinary tract typically host sarcomatoid carcinoma. This condition is exceptional in the head and neck area, specifically the larynx [5]. SpCC is a kind of tumor that has two distinct components: SCC, which can be either in situ or invasive, and SpCC, which has a sarcomatous appearance. SpCC is a type of cancer that originates from the squamous epithelium and contains a sarcomatous component that results from divergent mesenchymal development [6].

The cause of SpCC is still unknown, although it is strongly linked to a past history of smoking and alcohol use. Studies show a correlation between radiation exposure and SpCC, but factors like the amount and duration of exposure can complicate the accurate risk assessment [7]. Laryngeal carcinosarcoma is a rare disease, with a high male predilection (93%), a wide age range (24-81 years), and a median age at the presentation of 62.1 years. The disease is rare, with a mix of primary sites and stages (oral cavity, larynx, and paranasal sinuses). There is no clear consensus on its management and treatment, with conventional SCCs often requiring surgery, radiotherapy, or a combination of both [4]. SpCC has a higher prevalence in men (male-to-female ratio: 12:1). However, its incidence in females is on the rise, particularly in their sixth and seventh decades of life [6].

Laryngeal spindle cell (sarcomatoid) carcinomas (LSCSCs) have been a topic of discussion for years, with various terms applied to the confounding neoplasm. Over time, the medical community has come to recognize this unique, morphologically biphasic tumor process as a carcinoma with surface epithelial changes and spindle-shaped neoplastic proliferation. While the diagnosis of LSCSCs can be confidently established when the malignant surface epithelium is histologically evident, it becomes more difficult when the epithelium is ulcerated or denuded. Treatment modalities have not been critically assessed for their efficacy and long-term prognosis [8].

The tumor diagnosis includes histological and immunohistochemical examinations of epithelial and mesenchymal markers. Keratin, epithelial membrane antigens, KI, and K18 are epithelial indicators. Vimentin, desmin, S-100, osteopontin, and BMP are mesenchymal markers. Lewis et al. discovered that p53, a transcription factor that helps epithelial cells grow and differentiate, is especially useful for finding head and neck SpCC with epithelial tumor parts that are not fully differentiated [9]. The differential diagnosis includes several malignant spindle cell neoplasms, including fibrosarcoma, leiomyosarcoma, rhabdomyosarcoma, osteosarcoma, angiosarcoma, or Kaposi's sarcoma. Immunohistochemical tests are useful in these situations because they usually reveal the absence of epithelial marker expression in the tumor cells of the indicated sarcomas, thereby helping to exclude the diagnosis of sarcomatoid carcinoma [5].

The TNM classification method classifies SpCC based on the tumor's size and its potential to spread to other parts of the body. The location and stage of the tumor, along with criteria like the preservation of the larynx, the ability to manage voice after treatment, and a reduced risk of consequences, determine the choice of therapy. The majority of tumors are identified at an early stage, and the most successful methods for their removal include polypectomy and extensive local excision. Procedures such as local resection, partial laryngectomy, or complete laryngectomy can effectively treat tumors classified as stages 3-4. SpCC has a favorable five-year prognosis ranging from 65% to 95%. The indicators of adverse prognosis include advanced stages, large tumors, tumors beyond the voice cords, immobile vocal cords, prior radiation, and distant metastasis [1].

Treatment for glottic squamous cell carcinoma (LSCSC) focuses on achieving cancer cure, laryngeal preservation, good voice quality post-treatment, and low risk of complications. The two most important factors influencing management are tumor location and stage. After the initial biopsy, we can conservatively manage LSCSC with limited field irradiation, which provides 70-96% local control and voice preservation with the least likelihood of long-term complications. Radiation therapy alone can achieve 79-98% ultimate control of local disease when combined with salvage procedures for recurrent disease [10]. Zhang et al.'s study does not recommend radiotherapy as the sole treatment for laryngeal carcinosarcoma, but it can serve as a single treatment [11]. Given the high rates of local recurrence, mesenchymal tumor cells are not sensitive to radiation, and surgeons prefer surgical treatment [11]. However, Ballo et al.'s research suggests that radiation therapy can serve as an alternative therapy and effectively treat carcinosarcoma. Usually, mesenchymal tumor cells are considered to be resistant to radiation. Hence, the previously documented increased risks of local recurrence with radiation treatment lead to the choice of surgical treatment over radiation therapy [4].

In their study titled "Long-term results of RTOG 91-11," Forastiere et al. reported that patients never received chemotherapy as a single treatment or in combination with surgery or radiotherapy [12]. Factors influencing prognosis include tumor T stage, tumor location, vocal cord movement, and history of head and neck radiation therapy. Lymph node metastasis is not common in SpCC, but regional metastasis should be considered. The tumor's location is another important prognostic factor, with overall survival for supraglottic and glottic tumors of 91.7% and 69.3%, respectively [12]. Lei Wang et al.' study in April 2024 revealed that patients with sarcoma have a high expression of PD-L1, suggesting that sarcomas may respond well to immunotherapy. Currently, there are no recommendations that advocate the use of immunotherapy for advanced sarcoma. Anlotinib, a new tyrosine kinase inhibitor taken by mouth, has shown good clinical results in people with laryngeal sarcomatoid cancer (LSC) [13]. A phase II clinical study is now in progress to assess the impact of using a combination of camrelizumab and apatinib as the first therapy (NCT05265793).

While sarcotic carcinoma (SC) treatment efficacy is usually satisfactory, accurate biomarkers are needed to predict the efficacy of immunotherapy with immunomodulatory agents (ICIs). Current research hotspots include PD-L1 expression and TMB mutations. Studies show that PD-L1 expression increases PFS in SC treated with ICIs, with higher levels resulting in better efficacy. TMB is a marker for predicting ICI efficacy, with high TMB values resulting in a median OS of 18 months. However, the relationship between TMB and immunotherapy in SC has not been confirmed. Gene sequencing shows that 62% of patients with SC of HN have a CDKN2 mutation, potentially a positive biomarker for response to targeted CDK4/6 inhibitors. Multiple targeting drugs may improve long-term survival in HN's SC [14].

In our case, we administered conventional platinum-based chemotherapy to the patient, utilizing treatment approaches for carcinosarcomas observed in other sites and considering the patients' tolerance profile in the absence of immunotherapy options. The initial reaction was positive, with a median survival rate of one year. However, the patient's condition suddenly worsened, ultimately leading to her death. Based on our findings, we recommend further research into this subject to expand the body of knowledge and improve patient care.

## Conclusions

Carcinosarcoma is uncommon, malignant, and carcinomatous-sarcomatous. The varied language used to characterize these cancers makes it challenging to estimate their occurrence and obtain consensus on treatment. Sarcomatoid carcinoma, pseudosarcoma, and SpCC are the same tumors; however, carcinosarcoma is a biphasic neoplasm with malignant epithelial and mesenchymal components. Laryngeal carcinosarcoma may occur in both genders and has a similar clinical appearance to other laryngeal carcinomas. There is no consensus on appropriate laryngeal carcinosis treatment. The tumor stage, location, and size should dictate the surgical technique, although radiotherapy is not recommended due to the mesenchymal component's irradiation resistance. Frequent lymph node metastases necessitate neck dissection.

Carcinosarcoma of the larynx has a poorer prognosis than squamous cell laryngeal cancer, as well as more cervical lymph node metastases. We need to gain deeper insights into the biological functioning of these cancers to identify prognostic markers and develop effective treatment approaches. This unusual and challenging case of laryngeal sarcomatoid cancer with pulmonary metastases highlights the difficulties in its diagnosis and treatment. Multidisciplinary immunohistochemistry assays were required to differentiate this tumor from other sarcomas. The patient's limited drug tolerance, coupled with the absence of treatment guidelines, aggravated the situation. In these cases, the focus should be on early detection and comprehensive care.
